# Role of Nutraceuticals in COVID-19 Mediated Liver Dysfunction

**DOI:** 10.3390/molecules25245905

**Published:** 2020-12-13

**Authors:** Mohammed Sikander, Shabnam Malik, Anyssa Rodriguez, Murali M. Yallapu, Acharan S. Narula, Sanjaya K. Satapathy, Vijian Dhevan, Subhash C. Chauhan, Meena Jaggi

**Affiliations:** 1 Department of Immunology and Microbiology, School of Medicine, University of Texas Rio Grande Valley, McAllen, TX 78504, USA; mohammed.sikander@utrgv.edu (M.S.); fnu.shabnam@utrgv.edu (S.M.); anyssa.rodriguez01@utrgv.edu (A.R.); murali.yallapu@utrgv.edu (M.M.Y.); subhash.chauhan@utrgv.edu (S.C.C.); 2 South Texas Center of Excellence in Cancer Research, School of Medicine, University of Texas Rio Grande Valley, McAllen, TX 78504, USA; 3 Narula Research, LLC, 107 Boulder Bluff, Chapel Hill, NC 27516, USA; anarula1@nc.rr.com; 4 Division of Hepatology, Department of Internal Medicine, Sandra Atlas Bass Center for Liver Diseases and Transplantation, Barbara and Zucker School of Medicine, Northwell Health, Manhasset, NY 11030, USA; ssatapat@northwell.edu; 5 Department of Surgery, School of Medicine, University of Texas Rio Grande Valley, Edinburg, TX 78539, USA; vijian.dhevan@utrgv.edu

**Keywords:** COVID-19, coronavirus, ACE2, hepatic dysfunctions, hepatoprotective agents, nutraceuticals, SARS-CoV-2

## Abstract

COVID-19 is known as one of the deadliest pandemics of the century. The rapid spread of this deadly virus at incredible speed has stunned the planet and poses a challenge to global scientific and medical communities. Patients with COVID-19 are at an increased risk of co-morbidities associated with liver dysfunction and injury. Moreover, hepatotoxicity induced by antiviral therapy is gaining importance and is an area of great concern. Currently, alternatives therapies are being sought to mitigate hepatic damage, and there has been growing interest in the research on bioactive phytochemical agents (nutraceuticals) due to their versatility in health benefits reported in various epidemiological studies. Therefore, this review provides information and summarizes the juncture of antiviral, immunomodulatory, and hepatoprotective nutraceuticals that can be useful during the management of COVID-19.

## 1. COVID-19: From Outbreak to Pandemic

**Figure 1:**
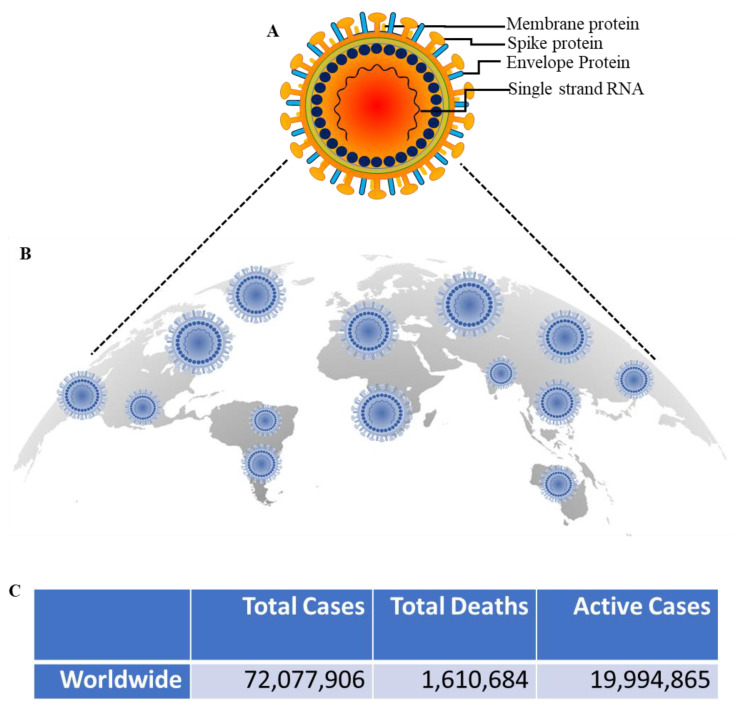
Global COVID-19 pandemic. (**A**); SARS-CoV-2 structure (**B**); Schematic representation of putative worldwide distribution of total cases, total deaths, and the active cases of COVID-19. (**C**); The number of total COVID-19 cases and deaths associated to COVID-19 was derived from Worldometer. Information obtained from https://www.worldometers.info/coronavirus/ on 12 December 2020.

The Coronavirus Disease 19 (COVID-19) is a highly infectious disease caused by a novel coronavirus, Severe Acute Respiratory Syndrome Coronavirus 2 (SARS-CoV-2) [1] (Figure 1A). The COVID-19 disease, which has infected millions and shocked the planet, emerges from the animal kingdom. Since December 2019, the first case of coronavirus disease (COVID-19) resulted from zoonotic transmission from the seafood and live wild animal market in Wuhan, China. As of 12 December 2020, approximately 72 million infections and more than 1,610,684 deaths have been reported, and these statistics are likely to be higher due to inadequate monitoring in parts of the world and asymptomatic carriers [2,3,4] (Figure 1B). The SARS-CoV-2 pandemic can be regarded as the largest global public health disaster since the onset of the 1918 pandemic. 

SARS-CoV-2 primarily causes respiratory symptoms, including flu-like symptoms and interstitial pneumonia, which may further progress into fatal Acute Respiratory Distress Syndrome (ARDS) condition [5,6]. However, other organs, particularly the liver, heart, and kidneys, are also affected, resulting in multi-organ failure and death in some patients [6]. Higher incidences of aminotransferases in and/or bilirubin were reported in COVID-19 patients to a varying degree. Moreover, several COVID-19 patients have experienced some type of liver injury, particularly those with serious or critical cases [7]. This prompted hepatologists to collaborate with other physicians, such as internal medicine and emergency departments, to protect the health status and avoid the adverse effects of COVID-19 in people with liver disease. Given the global burden of chronic liver disease, this pandemic could further worsen the treatment of patients at risk [8]. This review aims to gather information on medicinal plants and nutraceuticals with hepatoprotective activity, which can protect against the hepatic damage caused by COVID-19 and antiviral drugs. 

## 2. Pathogenesis of COVID-19

**Figure 2:**
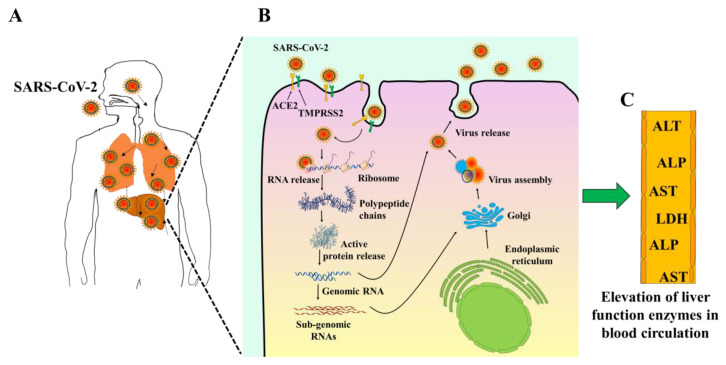
Schematic diagram showing the potential mechanisms of liver injury and abnormality in liver. (**A**); COVID-19 infection through the respiratory tract. (**B**); SARS-CoV-2 binds *via* its spike proteins to target cells receptor angiotensin-converting enzyme II (ACE2). (**C**); Liver function impairment with a mild-to-moderate increase of liver function enzymes level in the bloodstream.

Coronaviruses are single-stranded RNA viruses known to cause human respiratory tract or animal intestinal infection [9,10,11] (Figure 2A). Primarily, there are four main types, including α-coronavirus, β-coronavirus, δ-coronavirus, and γ-coronavirus [12]. Six coronaviruses, including SARS-CoV (Severe Acute Respiratory Syndrome CoV) and MERS-CoV (Middle East Respiratory Syndrome CoV), were reported to cause illness in humans prior to SARS-CoV-2 [13]. Similar to SARS-CoV and MERS-CoV, SARS-CoV-2 belongs to β-coronavirus. SARS-CoV-2 and SARS have a genome sequence homology of approximately 79% and is more closely related to SARS-like bat coronaviruses than SARS-CoV [14]. The spike protein recognizes and binds to the receptor and invades the host cell *via* clathrin-mediated endocytosis [15]. After internalization, the virus manipulates the cells’ reproductive machinery to produce more copies to infect other cells. SARS-CoV-2 also utilizes the cofactors, Furin and transmembrane proteases, serine 2 (TMPRSS2), protein cleaving enzymes, to cleave viral S-protein and further facilitate the virus-cell fusion [16,17]. As revealed in structure model analysis, SARS-CoV-2 binds to Angiotensin I Converting Enzyme 2 (ACE2), a host receptor, with more than a 10-fold higher affinity as compared to SARS-CoV [18] (Figure 2B). These findings explain the greater propagation capacity of SARS-CoV-2 in humans relative to SARS-CoV and the higher number of reported cases of COVID-19 relative to SARS-CoV infection [19]. The precise mechanism by which SARS-CoV-2 influences humans *via* S-protein binding to ACE2, the association intensity for the danger of human transmission, and how SARS-CoV-2 causes organ damage remains unclear, and further studies are required. 

## 3. Evidence for the Involvement of Liver in COVID-19 Infections

Early observational studies have shown the elevation in hepatic enzymes, including aspartate transferase, alanine transferase, and total bilirubin in COVID-19 patients [20,21,22,23,24,25,26,27] (Figure 2C). A study conducted by Chen et al. (2019) showed that more than a third of patients with COVID-19 have some liver function test abnormalities [25]. It is uncertain if these laboratory test variations are linked with a poorer prognosis. In another study of 1099 patients from 552 hospitals, Guan and colleagues found elevated AST levels in 112 (18.2 percent) patients with non-serious disease and 56 (39.4 percent) patients with severe disease [21]. In comparison, the proportion of pathological ALTs in severe cases (28.1%) was higher than in moderate cases (19.8%). Correspondingly, Huang et al. recorded that the proportion of ICU patients with liver damage (61.5%) was higher than non-ICU patients (25.0%) (25.0%) [24]. Recent clinical trials of COVID-19 suggest that elevated transaminases, elevated bilirubin, prolonged prothrombin period, hypoproteinemia, and intensity of blood test abnormalities can predict a worse outcome [28].

## 4. Mechanism of Liver Injury in COVID-19

**Figure 3:**
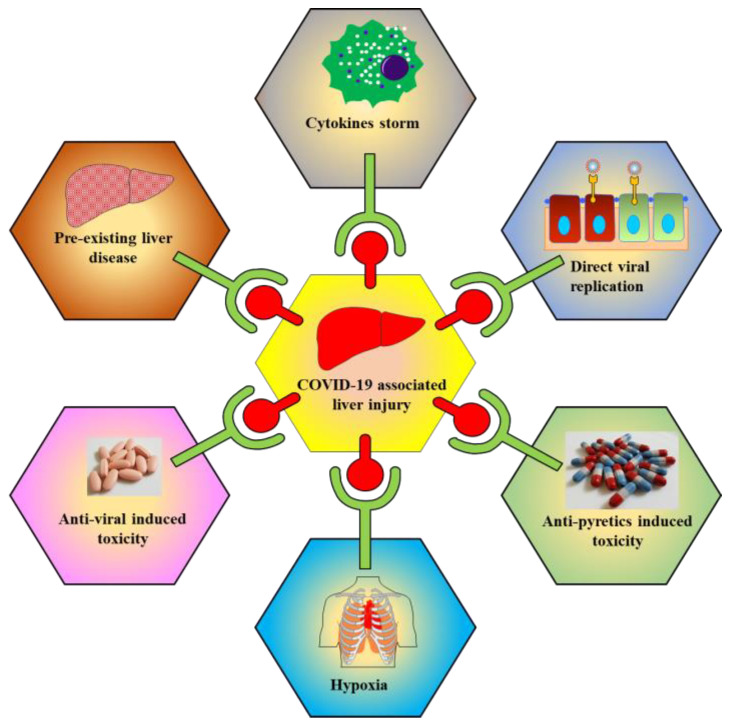
Schematic representations show multiple factors are responsible for COVID-19 associated liver toxicity/injury. Factors include cytokine storm, direct viral replication, antipyretics induced toxicity, hypoxia, antiviral induced toxicity, and pre-existing liver disease.

The exact mechanism of COVID-19 mediated liver injury is not fully known. In the following sections, various putative mechanisms involved in underlying hepatic injury are presented (Figure 3).

### 4.1. Direct Effect of COVID-19 on Liver

As previously discussed, SARS-CoV-2 uses the ACE2 receptor for entry into the host cells, where the lung is the main target for infection. Reports from RNA-seq analysis have confirmed the expression of the ACE2 receptor in the liver. Studies have shown the expression of ACE2 receptor on liver tissues [29]. Furthermore, liver histology of patients infected with COVID-19 reveals microvascular steatosis, multinuclear syncytial hepatocytes, and moderate lobular and portal activity [30]. Moreover, mitochondrial swelling, endoplasmic reticulum dilatation, glycogen degradation, and damaged cell membranes are demonstrated by electron microscopy. These microscopic and ultrastructural characteristics co-occur with the SARS-CoV-2’s cytopathic influence on hepatocytes, suggesting its role for the viral replication within liver cells [31].

### 4.2. Cytokine Storm Mediated Hepatic Damage

It is observed that the host body activates an immune response against SARS-CoV-2 infection to facilitate virus clearance and induce a sustained adaptive immune response. Moreover, serologic analysis has shown elevated levels of Th17, CD8+ T-cells, IL-2, IL-6, IL-10, TNF-α, GM-CSF, MCP-1, and macrophage inflammatory protein 1 α in patients with severe COVID-19 infection, as compared to those in control [24,32,33,34]. Understandably, these SARS-CoV-2 associated cytokine storms can damage many organs, including the liver and gut. Many patients eventually died following organ failure [35]. In a recent meta-analysis, the prevalence of chronic liver disease (CLD) patients (73 studies, 24,299 patients) was 3% among all COVID-19 patients [36]. The prevalence of CLD patients was similar in COVID-19 positive and negative populations (pooled OR 0.79 [95% CI 0.60, 1.05], *p* = 0.10). The presence of CLD was significantly associated with more severe COVID-19 infection (pooled OR 1.48 [95% CI 1.17, 1.87], *p* = 0.001) and overall mortality (pooled OR 1.78 [95% CI 1.09, 2.93], *p* = 0.02) [36].

### 4.3. Hypoxia Associated Liver Damage 

Extensive release of cytokines by the immune system in response to viral infection often results in sepsis symptoms, which caused mortality in 28% of COVID-19 cases [3]. Sepsis is generally referred to as the dysregulated immune response to an infection that results in multiple organ dysfunction [37]. The pathophysiology of sepsis-associated liver injury involves hypoxic liver injury due to several factors, including ischemia and shock, cholestasis, and overwhelming inflammation [38]. It is observed that sepsis is not uncommon in patients with existing liver cirrhosis [39], suggesting that pneumonia associated hypoxia is one of the most significant factors causing secondary liver injury in COVID-19 patients.

### 4.4. Antiviral Induced Hepatotoxicity

Microvascular steatosis and mild lobular and portal activities in postmortem biopsies reveal that liver injury is either caused by SARS-CoV-2 infection or drug-induced toxicity [30]. In another study, it was observed that antiviral therapies, including lopinavir/ritonavir used for the treatment of COVID-19, induces liver injury in patients [40]. Therefore, hepatic enzyme defects arose following the usage of a hepatotoxic medication; and antiviral induced damage may first be verified or removed.

### 4.5. Antipyretics Induced Hepatotoxicity

Many affected people were administered with antipyretic agents for fever relief during the COVID-19 outbreak. Many of these medicines include acetaminophen, a medication believed to inflict serious harm to the liver and/or trigger liver failure [30]. Acetaminophen is also known as APAP (acetyl-para-aminophenol) in the USA and paracetamol in Europe, is one of the most widely used antipyretics and analgesics medications in the world [41].

### 4.6. Pre-Existing Liver Disease Leads to Worst COVID-19 Outcome

Due to systemic immunodeficiency, patients with chronic liver disease and cirrhosis may have a higher risk of COVID-19 infections [42]. Moreover, a post-transplant patient is at higher risk due to immunosuppressive therapy [43,44]. However, the relationship between underlying liver disease and COVID-19 has not been fully studied. Patients with cirrhosis are at an increased risk of decompensation or development of acute-on-chronic liver failure when coupled with a bacterial, fungal, or viral infection [45,46]. Co-morbidities, including coronary artery disease, cerebrovascular disease, and chronic obstructive pulmonary disease, are more prevalent in hospitalized patients with severe/critical illnesses from COVID-19, and these patients are more likely to manifest abnormal liver chemistries [47]. Therefore, special attention should be paid to monitoring hepatic changes triggered by COVID-19 in patients with a pre-existing history of liver disease (especially older patients).

## 5. Hepatoprotective Agents

**Figure 4:**
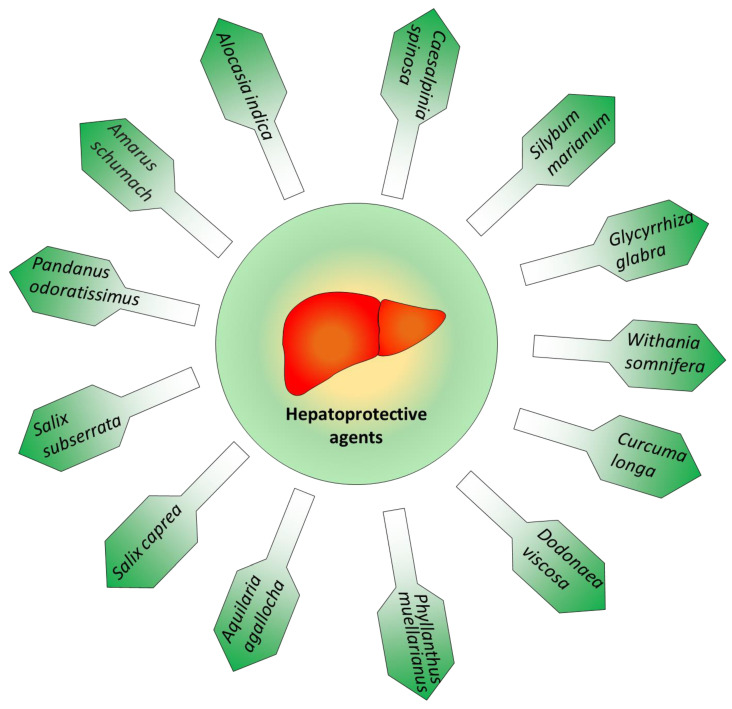
Various medicinal plants possessing potential hepatoprotective activities.

Most of the drugs are metabolized in the liver. As a result, liver injury can occur even though they are consumed for therapeutic purposes [48]. Since liver injury may result in fatty liver, hepatitis, fibrosis, cirrhosis, and cancer, this is considered as a serious health concern. Accumulated studies have reported that herbal compounds possess numerous medicinal properties. Natural products and nutraceuticals have shown potent therapeutic activity in liver injuries caused by several toxicants and drugs [49,50,51,52,53]. This section summarizes selective natural bioactive nutraceuticals exhibiting the hepatoprotective activity and pays specific attention to toxicants/antiviral induced liver injury (Figure 4). Few of these have shown potent antiviral activities and may be used in the future as possible options for the COVID-19 treatment 

### 5.1. Silybum Marianum

*Silybum marianum* belongs to the *Asteraceae* family and is native to the Mediterranean region. It is commonly known as milk thistle. This plant has thorny branches and milky sap, with oval leaves of up to 30 cm. The flowers are light pink in colors and can be up to 8 cm in diameter [54]. This plant is grown in Hungary, China, and South America. In Mexico, milk thistle is consumed as a nutritional substitute [55]. Silymarin is a naturally occurring compound found in *Silybum marianum* and is most notably composed of numerous flavolignans, including silybin, silydianin, and silychristine. Silybin constitutes approximately 50% to 70% of silymarin extracts. This plant possesses several pharmacological activities and is used to treat disorders related to the liver, gall bladder, and spleen [56]. Notably, because of its antioxidant, anti-inflammatory, and anti-fibrotic activities, silymarin is probably the most commonly used natural compound for hepatic disease care worldwide [57]. Most importantly, its medicinal property has been explored as hepatoprotective for supportive therapy in case of hepatic dysfunctions such as hepatitis, cirrhosis, and fatty liver [58,59]. Moreover, studies have shown that *Silybum marianum* is very potent against stress induced by toxicants, including poisonous mushrooms, alcohol, and toxic chemicals [60]. Bioactive components isolated from *Silybum* spp., silymarin, showed therapeutic benefits in acute and chronic viral, alcohol, and chemically induced hepatitis [61]. Silymarin is the most frequently used natural compound for treating hepatic diseases worldwide due to its antioxidant, anti-inflammatory, and anti-fibrotic activities. Inhibition of cyclooxygenase cycle, leukotrienes, and free radical production contribute to its cytoprotective effects in liver [62].

Silymarin has also been known to increase protein synthesis in hepatocytes [57]. Owing to its phenolic existence, it can give electrons to stabilize free radicals and reactive oxygen molecules [63]. Silymarin also modulates intracellular glutathione, which inhibits membranes from lipid peroxidation [64]. Silymarin also has antiviral effects as it affects RNA and DNA synthesis [65]. Eurasil 85 is a strong oral bioavailable silymarin formulation with potent antioxidant activity are found in clinical studies, and co-administration with several antiretrovirals has been shown to be safe [66,67]. Although the use of silymarin in the management of hepatic dysfunctions remains a historical interest, its utility in the management of SAR-COV-2 induced liver dysfunction should be explored [68].

### 5.2. Solanum Nigrum

*Solanum nigrum* is commonly known as “Black nightshade” and is often cultivated in open, wild temperate climate regions [69,70,71]. This also constitutes food crops in several developing countries [72]. *Solanum nigrum* has numerous medicinal properties [73]. In traditional medicine, plant leaves have reportedly been used to treat many illnesses, including seizures, asthma, nausea, ulcers, vomiting, diarrhea, some eye infections, and jaundice [74,75]. The extracts contain many polyphenolic compounds, such as phenolic acids and flavones [76]. This herb is used as a potential hepatoprotective agent [69,70,71]. It exhibits several antioxidant activities and is known to inhibit lipid peroxidation as a means for their mechanism of action [77,78]. Aqueous extract of *S. nigrum* has been shown to reduce hepatic enzymes ALT, AST, and ALP significantly. Moreover, this inhibits bilirubin’s level and scavenge the free radicals production, as observed in CCL_4_ induced hepatic damage in rats [79]. The antioxidant activity may be attributable to the polyphenolic compounds’ existence in stems and leaves [80].

### 5.3. Cichorium Intybus

The plant *Cichorium intybus,* commonly known as “chicory”, is indigenous to Western Asia, Egypt, North America, and Europe [81]. This displays several therapeutic properties including anti-microbial [82,83], immunomodulatory [84], antihepatotoxic [85,86,87] and anti-hypertiglycemia activities [88]. *Chichorium intybus* extract showed remarkable antioxidative effects *via* inhibition of thiobarbituric acid reactive substances production [89]. Notably, it has been used in many liver tonics for the ailment of the liver and digestive disorders [90]. The root extract of *C. intybus* has shown anti-hepatotoxic activity against CCL_4_-induced hepatic damage as demonstrated by decreased levels of aspartate aminotransferase (AST), alanine aminotransferase (ALT), and bilirubin in treated groups as compared to control groups [91]. Interestingly, Zhang et al. (2014) studied the anti-hepatitis activity of chicoric acid isolated from *Chichorium intybus* leaves and observed that it can block the viral protein and DNA synthesis [92].

### 5.4. Allium Sativum

*Allium sativum* has been used for more than 5000 years and is commonly known as garlic. *Allium sativum* is used as a spice and has numerous medicinal properties. The active components present in this plant are diallyl thiosulfinate and diallyl disulfide. Studies have reported that pretreatment with extracts restored antioxidant enzyme levels in GalN/LPS-treated hepatitis in animal models [93]. Furthermore, it is known to attenuate the nitric oxide-induced oxidative stress, and lipid peroxidation in CCL_4_ treated mice [94]. Garlic oil has also reduced serum AST, ALT, alkaline phosphatase (ALP), and gamma-glutamyl transferase (GGT) levels in CCL4-induced hepatotoxicity [95]. It has diverse pharmacological activities, including antibacterial, antiviral, antioxidant effects, anti-mutagenic, and immune-modulatory properties [96,97].

### 5.5. Glycyrrhiza Glabra

*Glycyrrhiza glabra,* commonly known as licorice, is native to the Mediterranean region, Western Asia and Europe. This plant is a very effective treatment for chest disease and asthma [56]. Studies have shown hepatoprotective activity of *G. glabra* in CCL_4_ induced hepatic damage. Following the oral administration, the level of hepatic iNOS, COX2, and TNF-alpha were significantly reduced in the treated group compared to the control group [98]. In another study, Kimura et al. showed that *G. glabra* is effective in attenuating the serum AST and ALT levels [99]. Glycyrrhizin, is a triterpenoid saponin, predominantly present in plant roots and possesses numerous pharmacological activities [100,101,102]. Interestingly, several studies have shown that glycyrrhizin exhibits potent activity against various human viruses [103,104,105,106,107,108,109,110,111,112,113,114,115,116]. Studies have shown the glycyrrhizin is used alone or in combination with other medications to combat coronavirus infections [117]. Recently, one study showed its ability to bind ACE2 that represses the SARS-CoV-2 receptor. Thus, targeting of ACE2 may also be very useful to prevent diffusion out of the virus from infected cells and invading new cells [118].

### 5.6. Phyllanthus Amarus

*Phyllanthus amarus* is a small plant and is commonly found in tropical regions and is used in many traditional medicines due to its diverse pharmacological activities [119]. Lignan phyllanthin, one of its essential bioactive constituents, has shown very potent activity as a hepatoprotective agent [120]. Histopathological analysis revealed that ethanolic extract of *P. amarus* attenuates the generation of intracellular ROS by enhancing the antioxidant levels against aflatoxin B_1_-induced hepatotoxicity [121]. Furthermore, an aqueous extract of *P. amarus* has been shown to inhibit HBV DNA polymerase activity as seen in in vitro experimental conditions, suggesting its potential use for viral infections in the liver [122]. Phytochemical screening of *P. muellarianus*, displayed the presence of several bioactive ingredients such as furosin, isoquercetin, phaselic acid, corilagin, nitidine, geranin, and gallic acid [123,124]. Aqueous extract of the *P. muellarianus* leaf has shown hepatoprotective activity against damage induced by p-acetaminophen in swiss albino mice [125]. It was observed that extract (*p* > 0.05) significantly attenuated acetaminophen-mediated alterations in ALT, alkaline phosphatase (ALP), AST, albumin (ALB), and total bilirubin (TB). Gallic acid, a well-known antioxidant agent, was documented to reverse AST, ALT, and ALP in acetaminophen-induced liver toxicity [126].

### 5.7. Withania Somnifera

*Withania somnifera* belongs to the family of *Solanaceae* and is commonly known as ashwagandha, Indian ginseng, or Winter cherry. Multiple parts of this medicinal plant, such as leaves, fruits, and stems, have therapeutic effects [127]. The popular bioactive ingredients present in this are withaferin A and withanolides. Several studies have shown the hepatoprotective activity of this plant without toxicity [127,128]. Withaferin A performs a crucial function as antiviral agents against several viruses, including HIV-1 [129], HPV [130], HSV [131], and infectious bursal diseases virus (IBDV) [131]. In a recent study, it has been reported that withaferin-A and withanone could bind and stably interact with the catalytic site of TMPRSS2, suggesting their use in the blocking of COVID-19 viral entry into host cells [132].

### 5.8. Curcuma Longa

*Curcuma longa* is a very famous spice native to India and Southeast Asia. This plant has diverse pharmacological activity, including antibacterial, antifungal, antiviral, and anticancer activities [133]. Curcumin (diferuloylmethane), a polyphenol present in this plant’s rhizome, is responsible for its therapeutic activity [134,135]. The hepatoprotective function of curcumin depends primarily on its strong anti-inflammatory and antioxidant effects. It also exhibits immunomodulatory activity by suppressing the production of cytokines IFN-gamma, ILs, and TNF-alpha. Curcumin is a very effective blocker of NF-kappa B and inhibits the synthesis of iNOS [136,137,138]. Research has also shown that curcumin inhibits hepatic stellate cell activation and collagen expression. Moreover, it has shown hepatoprotective effects in thioacetamide-induced liver injury and fibrosis [139]. Curcumin has also been reported to conduct antiviral activities against a large variety of viruses, including HIV-1, HSV-2, HPV and hepatitis virus [140,141].

### 5.9. Other Hepatoprotective Agents

*Capparis spinosa,* generally found in west and Central Asia, is commonly used as a cooking flavoring agent [142]. Several traditional medicines utilize this plant for the treatment of liver diseases [143]. This possesses numerous bioactivities, including antioxidant, anticancer, and antibacterial properties. It has been demonstrated that polyphenol present in the plant is responsible for its therapeutic activity. Liv-52, an Indian herbal preparation for liver disorders, also contains *Capparis spinosa,* an essential constituent [144]. Studies suggest that Liv-52 showed significant therapeutic effects in cirrhotic patients [144]. Additionally, the chemical constituent p-methoxy benzoic acid, from the aqueous extract of *C. spinosa,* expresses potent hepatoprotective activity against paracetamol and CCL_4_-induced hepatotoxicity [145].

*Aquilaria agallocha* has several properties, including pharmacological effects, and shows anticancer, antioxidant, anti-inflammatory, antidiabetic, analgesic, antipyretic, laxative, antidiabetic, antimicrobial, antibacterial, and anticonvulsant protective activities [146]. The hepatoprotective effects of the ethanolic extract of *A. agallocha* leaves in PCM-induced hepatotoxicity in Sprague–Dawley (SD) rats show a substantial decrease in AST, ALP, ALT, lactate dehydrogenase (LDH), CHL, TB, and an increase in ALB, total protein concentration, and prevention of PCM-induced histopathological changes in the liver [147].

*Dodonaea viscosa* belongs to the soapberry family and is widely distributed in the subtropical, warm, tropical, temperate regions of Africa, the Americas, Australia, and Southern Asia. Aqueous: methanolic (70:30) leaves extract of *D. viscosa* has shown to exhibit the hepatoprotective activity by reducing the serum level of TAG, total cholesterol, LDL-C, HDL-CHL, ALT, and AST compared to experimental control sample [148]. Another plant *Salix caprea,* referred to as goat willow, is a predominant species of willow in Europe and western/central Asia [149]. The detailed knowledge of various natural compounds’ hepatoprotective activity has been summarized in the review [150].

Ethanolic extract of *Salix subserrata* has shown significant hepatoprotective activity in CCl_4_-induced liver toxicity [149] by decreasing serum enzymes levels and reversing hepatic tissue damage caused by CCl_4_. Studies have shown that the hepatoprotective activity of ethanolic *Pandanus odoratissimus* roots extracts in PCM-induced hepatotoxicity in rats resulted in a substantial decrease in the higher levels of serum marker enzymes [151]. Another plant named *Alocasia indica,* commonly cultivated especially in West Bengal, Assam, Maharashtra, and Southern India, has been assessed for hepatoprotective activity in CCL_4_-induced hepatic injury in male Albino Wistar rats [152]. It has been reported that the antioxidant activity of this plant has the potential for designing drugs for liver diseases. *Opuntia ficus-indica,* another plant, is a cactus species that are commonly distributed in the arid and semiarid parts of the world and is believed to have originated from Mexico. Aqueous extract (2 mL/kg) from the cactus leaves (cladodes) has shown potent hepatoprotective activity in CCl_4_-induced toxicity in Wistar male rats by decreasing AST and ALT levels [153]. The use of these natural phytoagents or nutraceuticals alone or in combination may protect the liver and modulates coronavirus infection, and provide recommendations for further study.

## 6. Conclusions

The COVID-19 pandemic has resulted in a global crisis in public health. As noted in COVID-19 patients, liver injury is very common, caused by either direct or indirect damage to organs, including the overshooting inflammatory response. Importantly, the drug-induced liver injury should not be overlooked during coronavirus infection treatment and should be carefully studied. Ultimately, it is imperative to find alternative methods for hepatoprotection. This review provides information on the medicinal plants used for various hepatic disorders over several decades. It also highlights the pathways that these plant-based medicines can seek to reduce the burden of disease. The potential efficacy of these bioactive nutraceuticals should be explored in COVID-19 patients and at-risk populations.
